# Combinatorial Cytotoxic Effects of Damnacanthal and Doxorubicin against Human Breast Cancer MCF-7 Cells in Vitro

**DOI:** 10.3390/molecules21091228

**Published:** 2016-09-14

**Authors:** Muhammad Yusran Abdul Aziz, Nadiah Abu, Swee Keong Yeap, Wan Yong Ho, Abdul Rahman Omar, Nor Hadiani Ismail, Syahida Ahmad, Mehdi R. Pirozyan, Nadeem M. Akhtar, Noorjahan Banu Alitheen

**Affiliations:** 1 Department of Cell and Molecular Biology, Faculty of Biotechnology and Biomolecular Sciences, Universiti Putra Malaysia, Serdang 43400, Selangor, Malaysia; yusranaziz@gmail.com (M.Y.A.A.); nadyaboo@gmail.com (N.A.); 2 UKM Medical Molecular Biology Institute (UMBI), UKM Medical Centre, Jalan Ya’acob Latiff, Bandar Tun Razak, Cheras 56000, Kuala Lumpur, Malaysia; 3 Institute of Bioscience, Universiti Putra Malaysia, Serdang 43400, Selangor, Malaysia; skyeap2005@gmail.com (S.K.Y.); aro@upm.edu.my (A.R.O.); m_rasoli_p@yahoo.com (M.R.P.); 4 School of Biomedical Sciences, The University of Nottingham Malaysia Campus, Jalan Broga, Semenyih 43500, Selangor, Malaysia; WanYong.Ho@nottingham.edu.my; 5 Faculty of Applied Sciences, Universiti Teknologi Mara, Shah Alam 40450, Selangor, Malaysia; norhadiani@salam.uitm.edu.my; 6 Department of Biochemistry, Faculty of Biotechnology and Biomolecular Sciences, Universiti Putra Malaysia, Serdang 43400, Selangor, Malaysia; syahida@upm.edu.my; 7 School of Medical Sciences, University New South Wales, Wallace Wurth Building, Sydney, New South Wales 2052, Australia; 8 Faculty of Industrial Sciences and Technology, Universiti Malaysia Pahang, Lebuhraya Tun Razak, Kuantan 26300, Pahang, Malaysia; nadeemupm@gmail.com

**Keywords:** damnacanthal, doxorubicin, MCF-7, combination

## Abstract

Despite progressive research being done on drug therapy to treat breast cancer, the number of patients succumbing to the disease is still a major issue. Combinatorial treatment using different drugs and herbs to treat cancer patients is of major interest in scientists nowadays. Doxorubicin is one of the most used drugs to treat breast cancer patients. The combination of doxorubicin to other drugs such as tamoxifen has been reported. Nevertheless, the combination of doxorubicin with a natural product-derived agent has not been studied yet. *Morinda citrifolia* has always been sought out for its remarkable remedies. Damnacanthal, an anthraquinone that can be extracted from the roots of *Morinda citrifolia* is a promising compound that possesses a variety of biological properties. This study aimed to study the therapeutic effects of damnacanthal in combination with doxorubicin in breast cancer cells. Collectively, the combination of both these molecules enhanced the efficacy of induced cell death in MCF-7 as evidenced by the MTT assay, cell cycle, annexin V and expression of apoptosis-related genes and proteins. The effectiveness of doxorubicin as an anti-cancer drug was increased upon addition of damnacanthal. These results could provide a promising approach to treat breast cancer patients.

## 1. Introduction

Breast cancer is a complex disease that results from the interaction of multiple environmental, hormonal, and lifestyle risk factors with the individual’s genome [1]. Although the inherited risk factors cannot be changed, most lifestyle factors are modifiable and the risks for many women to get breast cancer can be reduced. Approximately, one third of the cancer in women arises from the breast. In 2007, there were 3242 female breast cancer cases reported in Malaysia, which accounted for 18.1% of all cancer cases registered and 32.1% of all female cases. Tamoxifen is the drug of choice in the treatment of estrogen receptor positive breast cancer [2]. Although chemoprevention using tamoxifen showed positive result in reduction of breast cancer patient’s mortality, women need to be informed of the adverse effects of tamoxifen. The adverse effects include vaginal discharge, hot flashes, and other significant risks for stroke (2-fold increased risk), endometrial cancer (2 per 1000 women per year), and life-threatening thromboembolic disease (2- to 3-fold increased risk) [3]. Although tamoxifen is the drug of choice in the treatment of estrogen receptor positive breast cancer, breast cancer patients are often treated with combinations of other drugs like doxorubicin for their treatment. Doxorubicin may be utilized after relapse in patients treated with tamoxifen or in patients with estrogen receptor negative breast cancer [4]. Doxorubicin is a powerful antitumoral drug and the effect of doxorubicin on tumor cells is mediated through multiple mechanisms. These include intercalation in DNA molecules, breaking of DNA strands by interaction with topoisomerase II, free radical formation and alteration of membrane structure [5]. Although tamoxifen and doxorubicin have been utilized in combination, the advantage of this combination in terms of therapeutic efficacy remains controversial. According to an in vitro by study Woods et al. [6], antagonistic effects between tamoxifen and doxorubicin were observed in the MCF-7 human breast cancer cell line. According to Woods et al. there are many factors that may influence the ultimate response to antitumor drugs in combination regimens like the clinical pharmacokinetics of the administered drugs, innate drug resistance and the extent of hormone dependency [6].

For centuries, scientists and medical professionals have been investigating chemical constituents in all parts of *Morinda citrifolia* (Noni or Yor). *Morinda citrifolia* L. (Rubiaceae) is a small tree, known commercially as noni that grows widely throughout the Pacific and is one of the most significant sources of traditional medicines among Pacific Islander societies in Hawaii, Fiji, Vanuatu, New Guinea, New Caledonia, and the Solomon Islands. All parts of the plant, including the roots, barks, stems, leaves, and fruits have been used traditionally as folk medicines for the treatment of many diseases, including diabetes, hypertension, and cancer [7]. According to Furusawa et al. [8] noni fruit juice is not cytotoxic in cell cultures (Lewis lung carcinoma cell line, sarcoma 180 cells, human KB carcinoma cell line, or normal NIH/3T3 and BALB/3T3 cell lines), but the juice can indirectly kill the cancer cells via activation of the cellular immune system involving macrophages, natural killer cells and T cells. Hence, noni fruit juice is one the powerful antitumor immunostimulators of plant food origin without having toxicity. Anekpankul et al. [9] reported that this plant contains several medicinally active components exhibiting various therapeutic effects. Roots of *Morinda citrifolia* are the source of important compounds, i.e., anthraquinones, which have been proven to have anti-viral, anti-bacterial, and anti-cancer activities. The most medicinally valuable anthraquinone in the roots of this plant is damnacanthal, which has been used for the treatment of chronic diseases such as cancer and heart disease [9].

It would be interesting if damnacanthal were used in combination with other drugs that are used in treating patients diagnosed with estrogen receptor positive breast cancer, since tamoxifen and doxorubicin shows antagonistic effects when used in combination on MCF-7 cells [6]. Although there are reports on the clinical application of noni juice as a supplemental agent for cancer treatment [10], there is no report on the effects of combining other anticancer drugs with damnacanthal. That study indicated that noni juice is able to enhance the therapeutic effect of the anticancer drug taxol on leukemia cells and this finding prompted the study on the use of a combination of anticancer drug with other phytochemical such as damnacanthal. Thus, the combination may be able to decrease the dose of synthetic anticancer drugs used, increase the tolerance of patients to the toxicity of anticancer drugs and increase the immune function. In this study, a combination of damnacanthal and doxorubicin were used to determine whether damnacanthal was able to enhance the therapeutic effect of doxorubicin or not. To achieve this objective, different concentrations of damnacanthal based on its CD_50_ value (the concentration that inhibited 50% viability of the MCF-7 cell population by the treatment) were used to treat MCF-7 cells with or without the presence of doxorubicin.

## 2. Results

### 2.1 Combination of Damnacanthal and Doxorubicin Decreased MCF-7 Cell’s Viability

**Figure 1:**
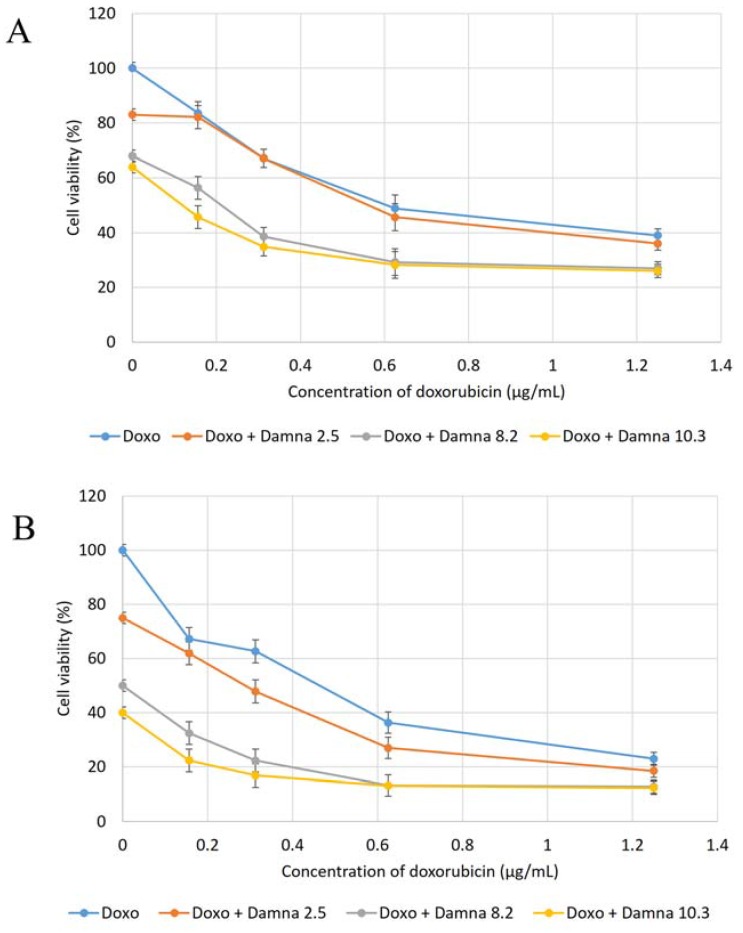

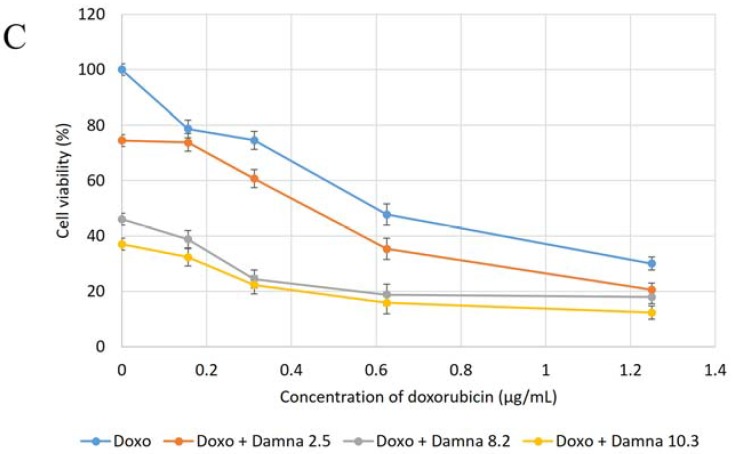
Cytotoxic effects of combination of doxorubicin and damnacanthal on MCF-7 after (**A**) 24 h, (**B**) 48 h and (**C**) 72 h incubation. Note: doxo: only treated with doxorubicin alone; doxo + damna 2.5: treated with various concentration of doxo in combination with fix dosage of damnacanthal at 2.5 µg/mL; doxo + damna 8.2: treated with various concentration of doxo in combination with fix dosage of damnacanthal at 8.2 µg/mL; doxo + damna 10.3: treated with various concentration of doxo in combination with fix dosage of damnacanthal at 10.3 µg/mL. Error bars represent the standard error of the mean.

Figure 1 presents the cytotoxic effects of doxorubicin and a combination of doxorubicin and damnacanthal on MCF-7 cells at 24, 48 and 72 h, respectively. From Figure 1, the CD_50_ value of doxorubicin on MCF-7 was estimated at around 0.55 ± 0.02 µg/mL. In Figure 1A, which was after 24 h incubation, the CD_50_ value dropped to less than 0.55 ± 0.02 µg/mL when MCF-7 cells were treated with a combination of doxorubicin and damnacanthal. The CD_50_ value dropped to 0.50 ± 0.03 µg/mL, 0.20 ± 0.02 µg/mL and 0.12 ± 0.05 µg/mL when MCF-7 cells were exposed to 2.5 ± 0.12 µg/mL, 8.2 ± 0.07 µg/mL and 10.3 ± 0.15 µg/mL of damnacanthal, respectively. The pattern is also the same in Figure 1B which is after 48 h of incubation. The CD_50_ value was reduced to less than 0.55 ± 0.02 µg/mL when MCF-7 cells were treated with a combination of doxorubicin and damnacanthal. The CD_50_ value was significantly reduced to 0.30 ± 0.12 µg/mL, 0.11 ± 0.01 µg/mL and 0.10 ± 0.03 µg/mL when MCF-7 cells were exposed to 2.5 µg/mL, 8.2 µg/mL and 10.3 µg/mL of damnacanthal, respectively.

Lastly in Figure 1C, which is after 72 h of incubation, the CD_50_ value also dropped to less than 0.55 ± 0.02 µg/mL when MCF-7 cells were treated a combination of doxorubicin and damnacanthal. The CD_50_ value significantly decreased to 0.45 ± 0.13 µg/mL, 0.12 ± 0.11 µg/mL and 0.10 ± 0.15 µg/mL when MCF-7 cells were exposed to 2.5 µg/mL, 8.2 µg/mL and 10.3 µg/mL of damnacanthal, respectively. From the results, the percentages of viability of MCF-7 cells after treatment with combination of doxorubicin and damnacanthal were decreased compared to the percentages of viability of MCF-7 cells when treated with doxorubicin alone. This indicates that damnacanthal can act in combination with doxorubicin.

### 2.2 Combination of Damnacanthal and Doxorubicin Altered the Morphological Appearance And Induced Apoptosis in MCF-7 Cells

**Figure 2:**
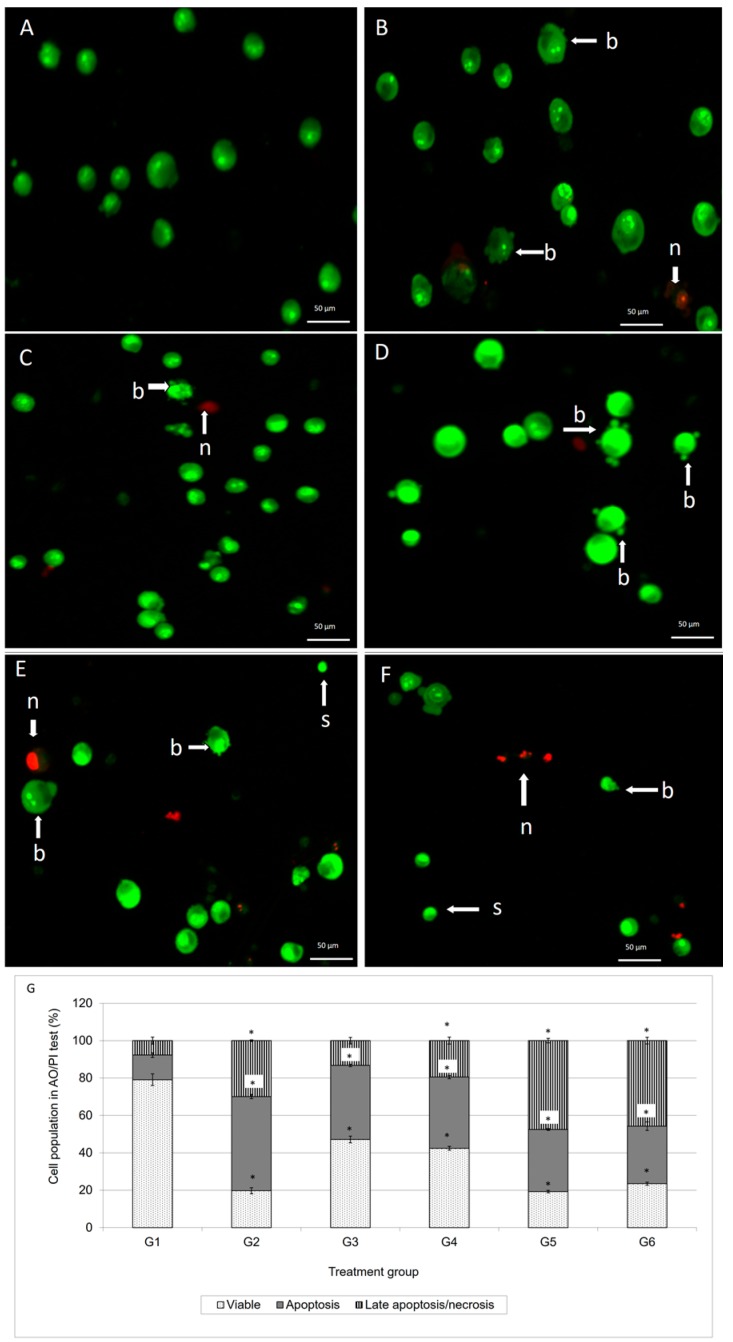
Fluorescent photomicrographs of MCF-7 cells treated with G1 (**A**); G2 (**B**); G3 (**C**); G4 (**D**); G5 (**E**) and G6 (**F**) at 72 h. Morphological changes following exposure to treatment are typical of apoptosis, showing s = cell shrinkage, b = membrane blebbing, and d = necrotic cells; (**G**) Population of viable, apoptosis and late apoptosis/necrosis of 200 cells randomly count for G1 to G4. G1: Control; G2: doxorubicin at 0.55 µg/mL: G3; damnacanthal at 2.5 µg/mL: G4; damnacanthal at 8.2 µg/mL: G5; doxorubicin at 0.4 µg/mL and damnacanthal at 2.5 µg/mL and G6; doxorubicin at 0.2 µg/mL and damnacanthal at 8.2 µg/mL. Each value represents the means ± S.E.M. for three assays in triplicate each. The differences between the control group and treated group were determined by one-way ANOVA (* *p* < 0.05).

AO/PI staining is considered the appropriate method for evaluating the changes of nuclear morphology and is used to quantify the cellular profile of viable, apoptotic and necrotic cells. Viable (green intact cells), apoptotic (green shrinking cells with condensed or fragmented nucleus) and late apoptotic and necrotic cells (red cells) were quantified from a population of 200 cells for the data to be statistically significant. The same procedure was carried out on cells treated for 72 h. Results were expressed as a proportion of the total number of cells examined. The experiment was repeated at least three times with triplicate samples for each experiment. The results of AO/PI staining is shown in Figure 2, which shows the fluorescence photomicrographs of MCF-7 cells after 72-h of treatment.

**Figure 3:**
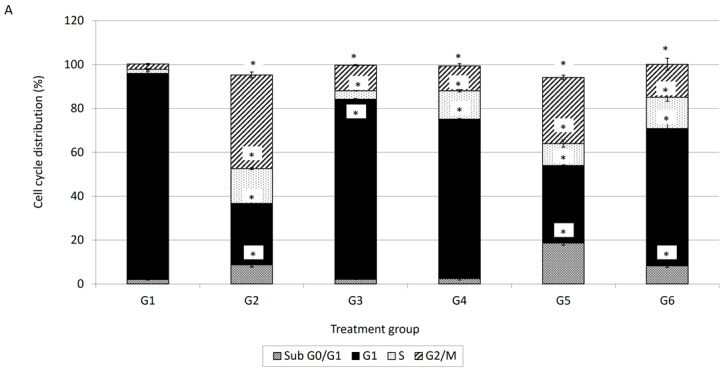

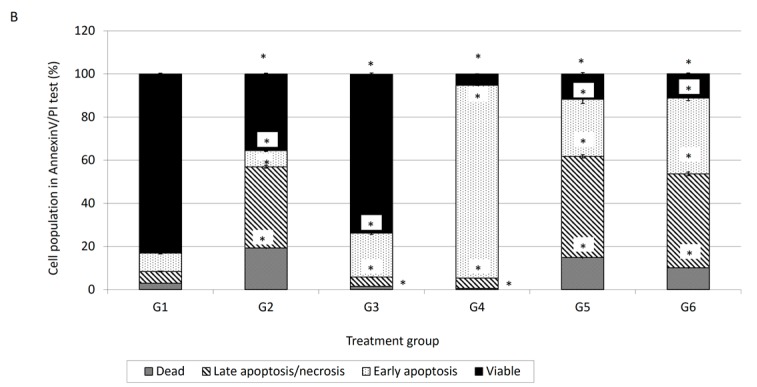
(**A**) Perturbations of cell cycle phase of MCF-7 cell after 72 h treatment; (**B**) FACS analysis of Annexin V and PI binding of MCF-7 cell after 72 h treatment Notes: G1: Control; G2: Doxorubicin at 0.55 µg/mL: G3; Damnacanthal at 2.5 µg/mL: G4; Damnacanthal at 8.2 µg/mL: G5; Doxorubicin at 0.4 µg/mL and Damnacanthal at 2.5 µg/mL and G6; Doxorubicin at 0.2 µg/mL and Damnacanthal at 8.2 µg/mL. Values within the same row and experiment having an asterisk indicate significance difference *(p* < 0.05*)* from the control groups. Error bars represent the standard error of the mean.

In comparison with the spontaneous cell death as observed in G1 (untreated control cells), substantial apoptotic cells were detected in MCF-7 cells treated with G2 (doxorubicin at 0.55 µg/ml), G3 (damnacanthal at 2.5 µg/mL), G4 (damnacanthal at 8.2 µg/mL), G5 (doxorubicin at 0.55 µg/mL and damnacanthal at 2.5 µg/mL) and G6 (doxorubicin at 0.2 µg/mL and damnacanthal at 8.2 µg/mL). Changes in the distribution of cell populations in various cell cycle phases after 72 h of MCF-7 cell treatment with G2 (doxorubicin at 0.55 µg/mL), G3 (damnacanthal at 2.5 µg/mL), G4 (damnacanthal at 8.2 µg/mL), G5 (doxorubicin at 0.55 µg/mL and damnacanthal at 2.5 µg/mL) and G6 (doxorubicin at 0.2 µg/mL and damnacanthal at 8.2 µg/mL) is displayed in Figure 3A. In comparison with the cell death as observed in G1 (untreated control cells), a significant (*p* < 0.05) amount of cells in sub-G_0_/G_1_ phase was detected in MCF-7 cells treated with G2 (doxorubicin at 0.55 µg/mL), G5 (doxorubicin at 0.55 µg/mL and damnacanthal at 2.5 µg/mL) and G6 (doxorubicin at 0.2 µg/mL and damnacanthal at 8.2 µg/mL). This sub-G_0_/G_1_ population is higher than the MCF-7 cells treated with G3 and G4 which are damnacanthal alone. The sub-G_0_/G_1_ population increased further from 2% for the control group to 8.7%, 18.7% and 8% after exposure to G2, G5 and G6, respectively, after 72 h. The percentage of sub-G_0_/G_1_ population are higher in G5 and G6 of MCF-7 cells which is the combination of doxorubicin and damnacanthal compared to the MCF-7 cells treated with either doxorubicin or damnacanthal alone. Apart from that, as shown in Figure 3A, there is cell cycle arrest in G_1_ phase and G_2_/M phase. The percentage of G_1_ phase was increased after MCF-7 cells were treated with G3 and G4, which is damnacanthal alone. Meanwhile the percentage of G_2_/M phase was increased in treatment G2 which is doxorubicin alone. After 72 h of incubation, the early apoptosis population of MCF-7 cells treated G2, G5 and G6 were decreased compared to 24 h of incubation time. However, the early apoptosis population of MCF-7 cells treated with G4 treatment is slightly increased.

While the early apoptosis population for MCF-7 cells treated with G2, G5 and G6 decreased, a pronounced increment in the double-positive (Annexin V^+^/PI^+^) late apoptosis population for MCF-7 cells was detected. The percentage of dead cell population also increased in G2, G5 and G6, as shown in Figure 3B.

### 2.3 Combination of Damnacanthal and Doxorubicin Regulated the Expression of Apoptotic-Related Genes and Proteins

In this study, expression levels of the genes listed in Table 1 were analysed using GeXP. Only those genes that showed detectable expression levels are displayed in the graphs. 

**Figure 4:**
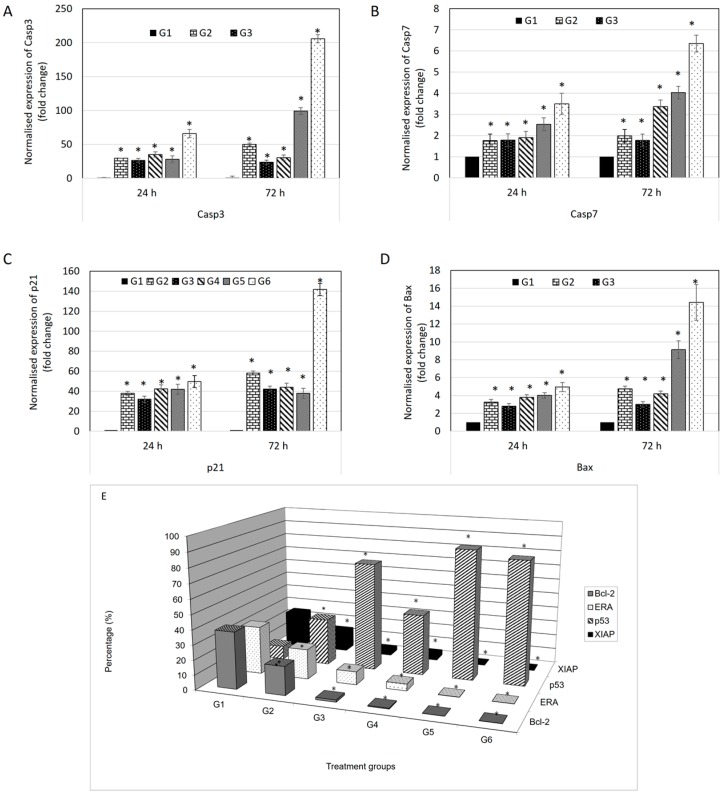
Normalised fold changes of (**A**) caspase 3; (**B**) caspase 7; (**C**) p21 and (**D**) Bax genes in MCF-7 cells after 24 and 72 h treatment; (**E**) Flow cytometry of protein expression of MCF-7 cell after 72 h treatment. Notes: G1: control; G2: doxorubicin at 0.55 µg/mL: G3; damnacanthal at 2.5 µg/mL: G4; damnacanthal at 8.2 µg/mL: G5; doxorubicin at 0.4 µg/mL and damnacanthal at 2.5 µg/mL and G6; doxorubicin at 0.2 µg/mL and damnacanthal at 8.2 µg/mL. Each value represents the means ± S.E.M. for three assays in triplicate each. The differences between the control group and treated group were determined by one-way ANOVA (* *p* < 0.05).

From Figure 4A,B, genes that showed significant changes in expression level included BAX, p21, caspase-3 and caspase-7. Figure 4A shows changes in the expression level of genes after MCF-7 cells were treated with G1: Control; G2: doxorubicin at 0.55 µg/mL: G3; damnacanthal at 2.5 µg/mL: G4; damnacanthal at 8.2 µg/mL: G5; doxorubicin at 0.4 µg/mL and damnacanthal at 2.5 µg/mL and G6; doxorubicin at 0.2 µg/mL and damnacanthal at 8.2 µg/mL for 12 h. Expression levels of all genes were found to be increased for treatment (G2, G3, G4, G5 and G6) as compared to control (G1). For p21 gene, all treatments exhibited a significant increase (*p* < 0.05) as compared to control (G1). Meanwhile, the expression level of caspase-7 was found to increase significantly (*p* < 0.05) in G5 and G6 treatments as compared to control. Figure 4B shows changes on the expression level of genes after MCF-7 cells were treated with G1, G2, G3, G4, G5 and G6 for 24 h. As at 12 h, all genes were found to have increased in expression level for treatment (G2, G3, G4, G5 and G6) as compared to control (G1). For p21 gene, only G6 was found to increase significantly (*p* < 0.05) as compared to control (G1). Meanwhile, the expression level of caspase-3 was found significantly increased (*p* < 0.05) in the G2, G4, G5 and G5 treatments. Expression level of caspase-7 increased significantly (*p* < 0.05) when the cells were treated with G4, G5 and G6.

In this study, expression levels of protein that are involved in apoptosis; Bcl-2, p53, ER-alpha and XIAP were evaluated using BD FACS Calibur multicolor flow cytometer (BD Biosciences, Franklin Lakes, NJ, USA). Figure 4C shows the percentage changes in expression level of protein after MCF-7 cells were treated with G1: control; G2: doxorubicin at 0.55 µg/mL: G3; damnacanthal at 2.5 µg/mL: G4; damnacanthal at 8.2 µg/mL: G5; doxorubicin at 0.4 µg/mL and damnacanthal at 2.5 µg/mL and G6; doxorubicin at 0.2 µg/mL and damnacanthal at 8.2 µg/mL for 72 h.

Percentage expression level of p53 protein increased significantly (*p* < 0.05) with treatments G2, G3, G4, G5 and G6 as compared to control G1. Meanwhile, percentage expression level of Bcl-2, XIAP and ER-alpha protein decreased significantly (*p* < 0.05) with treatments G2, G3, G4, G5 and G6 as compared to control.

## 3. Discussion

Doxorubicin is an anthracycline antibiotic, formed from natural products produced by species of the soil fungus *Streptomyces.* It is used in breast cancer chemotherapy. However, the therapy using doxorubicin is of limited use due to its serious side effects such as bone marrow and considerable cumulative cardiac toxicity [11]. Magrath [12] suggested that the conjugation of doxorubicin to a specific carrier molecule may reduce its side effects and also overcome drug-acquired tumor cell resistance. Therefore, this study aimed to investigate the potential cytotoxic effect of damnacanthal against MCF-7 cells and its interaction with doxorubicin. In the beginning, the cytotoxic effects of the combination of damnacanthal and doxorubicin on MCF-7 cells were determined using a slightly modified MTT assay. As demonstrated in Figure 4A–C, the MCF-7 cells were treated with anti-cancer drug doxorubicin alone and its combination with damnacanthal. The results showed that the CD_50_ value of doxorubicin toward MCF-7 cells dropped when the cells were treated in combination with damnacanthal. The concentrations of damnacanthal corresponding to its CD_25_ (2.5 µg/mL), CD_50_ (8.2 µg/mL) and CD_60_ (10.3 µg/mL) values were determined based on the reported results. From the results, the population of MCF-7 cells decreased more when it was treated with the combination of doxorubicin and damnacanthal compared to the use of doxorubicin alone. The combination of damnacanthal and doxorubicin thus led to an enhancement of the cytotoxicity of doxorubicin. In principle, this result is similar to the cytotoxic effect of mistletoe (*Viscum album* L.) extract combined with doxorubicin on Jurkat cells [13]. It was reported that the extract’s interaction with doxorubicin produced synergistic effects when used in combination.

Staining of apoptotic cells with fluorescent dyes such as AO and PI is considered the appropriate method for evaluating the changes in nuclear morphology [14]. This AO/PI staining primarily exhibits the entire aspect of cellular alterations, counting chromatin condensation and nuclear fragmentation [15]. As demonstrated in Figure 2, the AO/PI double staining procedure allowed us to distinguish several sub-populations of apoptotic cells from viable, early-membrane intact apoptotic and necrotic cell populations’ [16] nuclear morphology such as perinuclear chromatin condensation, membrane blebbing, cell shrinkage, nuclear collapse and eventual DNA fragmentation of MCF-7 treated cells, while untreated MCF-7 cells appeared to have bright green nuclei with intact structures. These features were seen in the MCF-7 treated-population as dense as green areas in the cytoplasms. Furthermore, AO is a nucleic acid selective fluorescent cationic dye that develops a protonated positive charge when it cross the plasma membrane of viable and early apoptotic cells and intercalates into DNA and RNA to produce green fluorescence [15,17]. Meanwhile, PI interacts with nucleic acids in cells with lysed membranes making them appear orange [15,17]. As shown in Figure 2, the MCF-7 cells treated with doxorubicin and its combination with damnacanthal underwent apoptosis, rather than necrosis. This suggested that their combination was able to enhance the cytotoxic effects on MCF-7 cells, probably causing apoptosis.

Cell cycle analysis is important for evaluating cell cycle parameters of surviving cells upon treatment or cell damage. When cells are damaged the cell cycle is interrupted at certain checkpoints and this prevents the normal cells from being transformed into cancer cells [18]. It is a simple method to estimate apoptosis because the DNA fragmentation is a hallmark of apoptosis caused by cellular endonuclease. Nuclei of apoptotic cells contain less DNA than healthy G1 cells; as a result, a sub-G_0_/G_1_ peak will observed before the G_1_ peak in the fluorescence histogram [18]. Therefore, cell cycle analysis has been carried out to evaluate the effect of damnacanthal, doxorubicin or their combination on MCF-7 cells. In conjunction with the decrement of percentage of viable cells in the MTT assay, cell cycle analysis showed an increment in the sub-G_0_/G_1_ population, indicating the occurrence of apoptosis in MCF-7 cells treated with the combination of damnacanthal and doxorubicin. Cell cycle analysis revealed that MCF-7 cells treated with damnacanthal have induced apoptosis and cell cycle is arrested at the G_1_ phase while MCF-7 cells treated with doxorubicin have induced apoptosis and cell cycle is arrested at the G2/M phase. This finding was similar to that of Ling et al. [19]. They reported that doxorubicin-induced cytotoxicity is cell cycle dependent and is mediated, at least in part, by disturbance of the regulation of p34cdc2/cyclin B1 complex, thus leading to G2/M phase arrest. Andreas et al. [20] reported that the cytotoxic effects on doxorubicin treated HepG2 cells are generally considered to be cell cycle specific where arrest occurs at the G2/M phase. In the present study, we found that the combination of damnacanthal and doxorubicin had a higher percentage of cells with hypo-diploid DNA content (sub-G_0_/G_1_). DNA histograms for MCF-7 cells treated with the combination of damnacanthal and doxorubicin exhibited a prominent decrease of the G_1_ phase cell population at 72 h of incubation.

The early apoptosis process involves the alteration of cell membranes. In normal living cells, phosphatidylserine (PS) is located on the inner cytoplasmic surface of cell membrane. While apoptotic cells will expose PS on their intact cell membrane’s surface [21,22] the PS externalization can be detected by annexin V-FITC conjugation [23]. With annexin V-FITC/PI, it is possible to detect live, viable cells (annexin V-FITC^−^/PI^−^), early apoptosis (annexin V-FITC^+^/PI^−^) and late apoptotic or necrotic cells (annexin V-FITC^+^/PI^+^) [17]. However, flow cytometry cannot distinguish between late apoptotic and necrotic cells. Morphological analysis by light microscopy and/or AO/PI fluorescence microscopy should resolve this problem. This annexin V-FITC/PI analysis revealed the percentage of different cell characteristics for MCF-7 cells treated with either damnacanthal and doxorubicin alone or a combination of both. In Figure 3B, the percentages of early apoptotic cells are relatively higher for damnacanthal and doxorubicin treatment rather than the combination of both treatments but the percentages of late apoptotic cells are higher for the combination of both treatments. Meanwhile, the percentages of late apoptotic cells are relatively higher for the combination of both treatments compared to damnacanthal or doxorubicin alone (Figure 3B). The percentages of early apoptotic cell population were relatively higher compared to those obtained via the AO/PI double staining method. This is due to fact that the annexin V-FITC/PI method detected the earlier stage of cell apoptosis before the plasma membrane lost its integrity. While, the AO/PI double staining method targeted later stages of apoptosis than tests based on nuclear morphology [16].

The expression of p21 gene is controlled by p53 protein. p21 is a potent cyclin-dependent kinase inhibitor (CKI) where it binds to and inhibits the activity of cyclin-CDK2 or -CDK1 complexes, and thus functions as a regulator of cell cycle progression at G_1_ use consistent nomenclature (All cell cycle gap phases were change to G_0_, G_1_, G_2_ to avoid confusion with the treatment groups) [24]. Besides that, p21 acts as the stopper in the cell cycle that allows DNA repair and/or synthesis [25] and also a promoter of cell death [26]. In the evaluation of protein expression using a BD FACS Calibur multicolor flow cytometer, p53 protein showed increments in all treatments compared to control. This explains why expression of p21 also increased in the multiplex gene expression profiler (GeXP) system (Beckman Coulter, Brea, CA, USA). Additionally, BAX is a protein of Bcl-2 family which serves a pro-apoptotic function [27]. Like p21 gene, the expression of BAX protein is also upregulated by the tumor suppressor protein p53. From the analysis, the expression levels of BAX are higher in all treatments compared to control. While BAX is a pro-apoptotic gene, Bcl-2 is an anti-apoptotic gene [28]. This explains why the expression of Bcl-2 protein is higher in the control group and it is effectively down-regulated in treatment in protein evaluation using flow cytometry. BAX and Bcl-2 are involved in mitochondria-mediated apoptosis [29]. Differences in expression levels between BAX and Bcl-2, can be an indicator of sensitivity to apoptotic inducers. Moreover, caspases are a family of cysteinyl aspartate-specific proteases which are central mediators of apoptotic and inflammatory pathways. Caspases are divided in two groups: initiator and effector caspases [30]. Initiator caspases are activated when they bind to adaptor molecules, resulted in activation of effector caspases. The initiator caspase responsible for the mitochondrial pathway (intrinsic pathway) is caspase-9, while the initiator caspases for the transmembrane pathway (extrinsic pathway) are caspase-8 and -10. Both of these pathways share the effector caspases which are caspases-3, -6 and -7 [30]. After up-regulation of BAX protein, the outer mitochondrial membrane becomes permeable; inducing the leakage of pro-apoptotic molecules include cytochrome c, Smac/DIABLO, HtrA2/Omi, apoptosis inducing factor (AIF) and endonuclease G (Endo G) from the mitochondrial intermembrane space [31]. Cytochrome c induces the oligomeraization of apoptosis protease activating factor-1 (Apaf-1) [32]. Apaf-1 then recruits procaspase-9 molecules in a complex called the ‘apoptosome’ resulted in autoactivation of procaspase-9 [33]. The release of mature caspase-9 activates downstream caspase cascades include caspase-3 and -7 [34].

## 4. Materials and Methods

### 4.1. Reagents and Chemicals

3-(4,5-Dimethylthiazol-2-yl)-2,5-diphenyl tetrazolium bromide (MTT), RPMI-1640, phosphate buffer saline (PBS), bovine serum albumin (BSA) and ethylenediaminetetraacetic acid (EDTA) were all purchased from Sigma (Sigma-Aldrich, St. Louis, MO, USA). Foetal bovine serum (FBS) was purchased from PAA (GE Healthcare Bio-Sciences, Pittsburgh, PA, USA) and was heat-activated for 30 min in a 56 °C water bath prior to use. Tryple E from Gibco Invitrogen (Gibco, Invitrogen, Thermo Scientific, Hudson, NH, USA), DMSO (Thermo Scientific, Hudson, NH, USA). 96-well and 6-well plates, FITC-conjugated Annexin V kit were purchased from (BD Biosciences, San Jose, CA, USA).

### 4.2. Cell Viability Assay

The effect of the combination of damnacanthal and doxorubicin treatment on cell viability of MCF-7 cells was determined using a colorimetric technique, which is an MTT assay on a 96-well plate (BD) in RPMI 1640 media with 10% of FBS per well at cell density 1 × 10^5^ cell/wells incubated for 24 h, 48 h and 72 h (37 °C, 5% CO_2_ and 95% humidity). One hundred µL of diluted doxorubicin at 10 µg/mL was added into row A and row B. A series of two-fold dilution of extract was carried out down from row B until row G. The row H was left untouched and the excess solution (100 µL) was discarded and 100 µL of diluted compound (at concentration CD_25_: 2.5 µg/mL, CD_50_: 8.2 µg/mL and CD_60_: 10.3 µg/mL of damnacanthal towards MCF-7 cells after 48 h of incubation obtained from MTT results) was added into wells in the 96-well plate. Finally, the plate was read at 570 nm and 630 nm as reference wavelength by using a µ Quant ELISA Reader (Bio-tek Instruments, Maharashtra, India). The experiment was repeated for at least three times with triplicate samples for each experiment. The percentage of proliferation was calculated using the following formula:

Percentage of cell viability = (OD_sample_/OD_control_) × 100% (1)

### 4.3. Fluorescent Microscopy using Acridine Orange/Propidium Iodide (AO/PI) Double Staining Assay

MCF-7 cells (5 × 10^5^ cells) were seeded in 6-well plates and incubated at 37 °C in a 5% CO_2_ atmosphere. Twenty four h later, the medium in each well was removed and replaced with the various treatments (G1; control, G2; doxorubicin at 0.55 µg/mL, G3; damnacanthal at 2.5 µg/mL, G4; damnacanthal at 8.2 µg/mL, G5; doxorubicin at 0.55 µg/mL and damnacanthal at 2.5 µg/mL and G6; doxorubicin at 0.2 µg/mL and damnacanthal at 8.2 µg/mL). The concentration of doxorubicin at 0.55 µg/mL was obtained from the CD_50_ value of doxorubicin toward MCF-7 cells and the concentration of doxorubicin at 0.2 µg/mL was obtained from the CD_25_ value of doxorubicin towards MCF-7 cells. Meanwhile, the concentrations of damnacanthal used were same as in the cell viability assay described in Section 4.2. The plates were incubated at 37 °C in an incubator with 5% CO_2_ and 90% humidity for 24 and 72 h. After 24-h incubation, detached and anchored cells in the medium were collected. Anchored cells were detached with the use of tryple E (Gibco, Invitrogen, Thermo Scientific, Hudson, Waltham, MA, USA). The cell suspension was washed with PBS. Ten µL of the cells were then put on a glass slide and mixed with 10 µL of acridine orange (50 µg/mL) and propidium iodide (50 µg/mL) at a ratio of 1:1 in 1 mL of cells. Within 30 min, the slide was analyzed using fluorescent microscope (Nikon, Tokyo, Japan) using a combination of excitation and barrier filters at 450–490 nm and long pass filter.

### 4.4. DNA Cell Cycle Analysis

The perturbation in the distribution of cells in the different phases of the cell cycle was determined by flow cytometry. 5 × 10^5^ of MCF-7 cells were seeded into each well of a 6-well plate and incubated at 37 °C in 5% CO_2_ atmosphere. Twenty-four h later, the medium in each well was removed and replaced with the various treatments (G1; control, G2; doxorubicin at 0.55 µg/mL, G3; damnacanthal at 2.5 µg/mL, G4; damnacanthal at 8.2 µg/mL, G5; doxorubicin at 0.55 µg/mL and damnacanthal at 2.5 µg/mL and G6; doxorubicin at 0.2 µg/mL and damnacanthal at 8.2 µg/mL). The plate was incubated at 37 °C in an incubator with 5% CO_2_ and 90% humidity for 24 and 72 h. After the corresponding period, the samples were washed and transferred to a centrifuge tube (BD Biosciences). The cells were pelleted and fixed with 80% ethanol and incubated at 4 °C for 2 h. Then, the cells were re-pelleted and washed twice with PBS-sodium azide-EDTA buffer. The cell pellet was finally dissolved and stained in PBS buffer consisting of 0.1% Triton X-100, 10 mM EDTA, 50 µg/mL RNase and 2 µg/mL propidium iodide (PI) in the dark. The cell pellet was then incubated for half an hour in 4 °C prior to analysis by FACS-Calibur flow cytometer (BD Bioscience, San Jose, CA, USA). The analysis of stained cells by flow cytometer was conducted within 24 h. The experiment was repeated for three times with triplicate samples for each experiment.

### 4.5. Annexin V Binding Assay using Flow Cytometry 

5 × 10^5^ of MCF-7 cells were seeded into each well of a 6-well plate. After incubation for 24 h the cells were treated with various treatments (G1; control, G2; doxorubicin at 0.55 µg/mL, G3; damnacanthal at 2.5 µg/mL, G4; damnacanthal at 8.2 µg/mL, G5; doxorubicin at 0.55 µg/mL and damnacanthal at 2.5 µg/mL and G6; doxorubicin at 0.2 µg/mL and damnacanthal at 8.2 µg/mL) were added. Group treatments used were same as in the AO/PI staining assay (Section 4.3). The plate was incubated at 37 °C in an incubator with, 5% CO_2_ and 90% humidity for 24 and 72 h. After the corresponding incubation, cells were harvested, washed and stained with Annexin V-FITC (BD Biosciences) with propidium iodide (PI). The procedures were carried out according to the instructions provided by the manufacturer. Briefly, the culture was washed with PBS and re-suspended in 500 µL total volume that contain 100 µL of cells (5 × 10^5^ cells/mL), 10 µL PI (Sigma), 5 µL AnnexinV-FITC (BD Bioscience) and 400 µL binding buffer (BD Bioscience). After 15-minute incubation in dark, the cells were analyzed with FACS-Calibur flow cytometer (BD Biosciences). The experiment was repeated for three times with triplicate samples for each experiment.

### 4.6. Cell Treatment, RNA Extraction and cDNA Conversion

The MCF-7 cells were treated with with various treatments (G1; control, G2; doxorubicin at 0.55 µg/mL, G3; damnacanthal at 2.5 µg/mL, G4; damnacanthal at 8.2 µg/mL, G5; doxorubicin at 0.4 µg/mL and damnacanthal at 2.5 µg/mL and G6; doxorubicin at 0.2 µg/mL and damnacanthal at 8.2 µg/mL). Meanwhile untreated (G1) was used as controls. The expression profiles were analyzed at two time points, 12 and 24 h. After the treatment period, cells were trypsinized and washed twice with PBS in order to prepare for RNA extraction. Total RNA was extracted from the treated and untreated cell lines for both time points using the RNeasy® Mini Kits (Qiagen, Valencia, CA, USA). The eluted RNA was kept in −80 °C for future use. After RNA extraction and quantification, RNA from each sample was reverse transcribed to cDNA using the GenomeLab™ GeXP Start Kit (Beckman Coulter, Brea, CA, USA). The RT reaction then was run in a thermal-cycler (PTC-225, MJ Research, Watertown, MA, USA) with the following program: 48 °C for 1 min; 37 °C for 5 min; 42 °C for 60 min; 95 °C for 5 min; hold at 4 °C.

### 4.7. Polymerase Chain Reaction (PCR) and GenomeLab GeXP Genetic Analysis System

Primers were designed by importing the target gene ID or sequence into the eXpress Designer module of the eXpress Profiler (Table 1). The amplified products were designed to generate gene fragments with lengths between 150–350 nucleotides. An aliquot (9.3 μL) of the RT reaction was then transferred to the PCR reaction mix which contains MgCl_2_, the gene-specific forward chimeric primer plex, fluorescently-labeled universal forward primer, unlabeled universal reverse primer and Thermo-Start^®^ DNA polymerase (ABgene Thermo Scientific, Hudson, NH, USA) (Table 1). The 96-well plate containing the PCR reaction mixture was transferred to a thermal-cycler (PTC-225, MJ Research) and run under the following program: 1 cycle of 95 °C for 10 min followed by 35 cycles of 94 °C 30 s, 55 °C 30 s, 68 °C 1 min; hold at 4 °C. The level of the gene expression profiles of MCF-7 treated with doxorubicin and damnacanthal alone and combination at different time point (12 and 24 h) was assessed using GeXP. Subsequently, completed PCR product was mixed with 37.5 µL sample loading solution (SLS), 0.5 µL the DNA size standard-400 (GenomeLab™ GeXP Start Kit, Beckman Coulter, Brea, CA, USA) and overlaid with a layer of mineral oil before the sample was transferred to CEQ 8000 Genetic Analysis System (Beckman Coulter). By referring to the Frag-3 protocol, PCR products with fluorescently-labeled fragment were separated by 50 °C capillary gel electrophoresis according to their product size at 6.0 kV for 35 min, right after they undergo denaturation at 90 °C for 120 s and injection for 30 s at 2.0 kV. The raw data of separated product was first analyzed with the Fragment Analysis module of GenomeLab GeXP™ system software (version 10.2, Beckman Coulter, Brea, CA, USA). Then the information related to fragment of product, the height and area of the peak was imported into express Analysis module of the express Profiler software (version 8.0, Beckman Coulter, Brea, CA, USA) for analysis. With this software, housekeeping genes used in this experiment were checked for their consistency. GADPH was selected as reference gene for normalizing all data of targeted genes.

### 4.8. Flow Cytometry Intracellular Protein Detection

5 × 10^5^ of MCF-7 cells were seeded into each well of a 6-well plate and after incubation for 24 h the cells were treated with various treatments (G1; control, G2; doxorubicin at 0.55 µg/mL, G3; damnacanthal at 2.5 µg/mL, G4; damnacanthal at 8.2 µg/mL, G5; doxorubicin at 0.4 µg/mL and damnacanthal at 2.5 µg/mL and G6; doxorubicin at 0.2 µg/mL and damnacanthal at 8.2 µg/mL). The plate was incubated at 37 °C in an incubator with 5% CO_2_ and 90% humidity for 72 h. After the corresponding incubation, cells were harvested and washed. The fixation and permeabilisation was done by using BD Cytofix/Cytoperm^TM^ Fixation/Permeabilisation (BD Bioscience). Briefly, the cells were resuspended thoroughly and 250 µL of Fixation/Permeabilisation solution were added into each tube and incubated for 20 min at 4 °C. After 20 min, the cells were washed twice by 1 × BD Perm/Wash™ buffer. For each treatment, the cells were divided into 4 centrifuge tube (BD Bioscience). Then, the cells were stained with fluorochrome-conjugated monoclonal anti-mouse p53 and Bcl-2 (Santa Cruz Biotechnology, Inc., Dallas, TX, USA), anti-mouse XIAP (BD Biosciences) and anti-mouse estrogen receptor alpha (ER α) (Abcam, Cambridge, UK). The cells were washed twice with PBS to remove non-specific binding materials. The cells were stained again with secondary fluorochrome-conjugated monoclonal antibody mouse anti-p53, anti-Bcl2, anti-XIAP and anti-ER α (Abcam, Cambridge, UK). After that, the cells were washed twice to remove non-specific binding. Finally, the cells were analyzed by BD FACS Calibur multicolor flow cytometer (BD Biosciences).

### 4.9. Statistical Analysis

Results were expressed as Mean ± Standard Error (S.E.M). Differences between means were evaluated using ANOVA test (one way) followed by Tukey’s test and (*p* < 0.05) was taken as statistically significant.

## 5. Conclusions

In conclusion, the present results indicate that damnacanthal is effective in sensitising MCF-7 cells to doxorubicin treatment. Damnacanthal at IC_25_ and IC_50_ levels was able to reduce the IC_50_ value of doxorubicin to MCF-7 cells from 5.5 to 4.0 and 2.0 µg/mL, respectively. Based on the results from a cell viability assay, DNA cell cycle analysis and annexin V-FITC/PI staining, this natural product and drug combination targets MCF-7 cells via induction of apoptosis. Moreover, gene expression and protein detection show that this natural compound-drug combination, particularly at 0.2 µg/mL of doxorubicin and 8.2 µg/mL of damnacanthal, activated the apoptosis-related genes the most comparing to the other treatment groups. Additional in vivo studies on the combination of damnacanthal and doxorubicin are however needed to clarify the efficacy and safety of damnacanthal-doxorubicin in cancer treatment.

## Figures and Tables

**Table 1:** Details of the primer sequences used in GeXP study.

Gene	Accession Number	Product Size	Left Sequence	Right Sequence
BCL2	M14745	157	ACCACTAATTGCCAAGCACC	TTTTCCATCCGTCTGCTCTT
Fas	NM_000043	165	CTCCAAGGGATTGGAATTGA	TGCAGTCCCTAGCTTTCCTT
TNF alpha	NM_000594	171	CTATCTGGGAGGGGTCTTCC	ATGTTCGTCCTCCTCACAGG
Caspase3	NM_004346	182	GAACTGGACTGTGGCATTGA	ACCAGGAGCCATCCTTTGA
p21Cip1	NM_000389	202	TGTGGACCTGTCACTGTCTTG	TAGGGCTTCCTCTTGGAGAA
Cyclin A2	NM_001237	212	TATTGCTGGAGCTGCCTTTC	CTTTTCTCTTATTGACTGTTGTGCAT
BCL2L1	NM_001191	232	CCACAGCAGCAGTTTGGAT	GGGATTGTTCCCATAGAGTTCCACAA
MDM2	NM_002392	239	GGTGGGAGTGATCAAAAGGA	ACCAGGCTTTCATCAAAGGAA
Caspase7	NM_033340	247	CAGACCGGTCCTCGTTTGTA	ACCTCGGCATCTTTGTCTGTT
GAPDH ^a^	NM_002046	275	AAGGTGAAGGTCGGAGTCAA	AGATCTCGCTCCTGGAAGATG
CDK2	NM_052827	285	TGGTGGCGCTTAAGAAAATC	ACAGCTGGAACAGATAGCTCTTGA
ACTB ^a^	NM_001101	295	CTGGCACCACACCTTCTACA	AAGGGCATACCCCTCGTAGAT
Bax	BC014175	316	CCCTTTTGCTTCAGGGTTTC	ACAAAGTAGAAAAGGGCGACAA
KAN ^b^	Kan(r)	325	ATCATCAGCATTGCATTCGATTCCTGTTTG	AATTCCGACTCGTCCAACATC
Caspase9	NM_001229	332	GGGCTCACTCTGAAGACCTG	ATCTGGAAGCTGCTAAGAGCC
P53	NM_000546	340	TTTTGGGTTTTGGGTCTTTG	ATTCAACATGAGGGACAGCTT

^a^ Housekeeping genes used for normalization; ^b^ Internal control [35].
